# Low temperature magneto-morphological characterisation of coronene and the resolution of previously observed unexplained phenomena

**DOI:** 10.1038/srep38696

**Published:** 2016-12-07

**Authors:** Jason Potticary, Rebecca Boston, Liana Vella-Zarb, Alex Few, Christopher Bell, Simon R. Hall

**Affiliations:** 1 Complex Functional Materials Group, School of Chemistry, University of Bristol, Bristol, BS8 1TS, United Kingdom; 2 Department of Materials Science and Engineering Sir Robert Hadfield Building, Mappin Street Sheffield S1 3JD, United Kingdom; 3 Department of Chemistry, Durham University, Lower Mountjoy, South Road, Durham, DH1 3LE, United Kingdom; 4 Department of Chemistry, University of Malta, Msida, MSD2080, Malta; 5 School of Physics, HH Wills Physics Laboratory, Tyndall Avenue, Bristol, BS8 1TL, United Kingdom

## Abstract

The polyaromatic hydrocarbon coronene has been the molecule of choice for understanding the physical properties of graphene for over a decade. The modelling of the latter by the former was considered to be valid, as since it was first synthesised in 1932, the physical behaviour of coronene has been determined extremely accurately. We recently discovered however, an unforeseen polymorph of coronene, which exists as an enantiotrope with the previously observed crystal structure. Using low-temperature magnetisation and crystallographic measurements, we show here for the first time that the electronic and magnetic properties of coronene depend directly on the temperature at which it is observed, with hysteretic behaviour exhibited between 300 K and 100 K. Furthermore we determine that this behaviour is a direct result of the appearance and disappearance of the newly-discovered polymorph during thermal cycling. Our results not only highlight the need for theoretical models of graphene to take into account this anomalous behaviour at low temperatures, but also explain puzzling experimental observations of coronene dating back over 40 years.

Coronene (C_24_H_12_) is a highly crystalline polyaromatic hydrocarbon (PAH) found commonly as yellow, needle-like crystals. It is an ortho- and peri-fused system of seven, six-membered rings (Fig. 1). Its disc-shape and planar structure give it high symmetry (point group D_6_h) which, coupled with the 24 electron π-system of the sp^2^ carbon atoms, makes it an ideal model system for the study of the physical properties of graphene^1^,^2^,^3^,^4^,^5^,^6^,^7^,^8^,^9^. The use of coronene in this way is due in no small part to the fact that its physical properties under a wide range of conditions are well-known and predictable. Computationally, Density Functional Theory (DFT) has long made use of this predictability in order to calculate various physical parameters such as the cohesive energy, equilibrium cell volume, total electron density and electronic band structure of crystalline coronene^10^. These data also guide further experiments and predict results in fields as diverse as molecular electronics^11^, organic superconductors^12^ and astrophysics^13^,^14^. One reason for the high confidence in the observation and prediction of coronene’s properties was the fact that in the solid state, coronene exhibited no polymorphism. The crystal structure^15^ is monoclinic, with space group *P*2_1_*/a* (β = 110.9°, *a* = 16.11 Å, *b* = 4.70 Å, *c* = 10.10 Å and *Z* = 2) and can be considered to have a γ-herringbone structure (γ-coronene), Fig. 1(a). It was known that γ-coronene could be forced to form other polymorphs, but only at extremes of pressure^16^. What was perplexing, however, was that at low temperatures and ambient pressure, unaccountable experimental results were obtained. These anomalies were first observed in 1972, when solutions of coronene in organic solvents measured at 77 K showed inexplicable multiplicities in the absorption and fluorescence spectra^17^. Later work on the comparison of the luminescence spectra of coronene at 298 K and at 90 K under ambient pressure revealed a large difference in spectral shape^18^. Aside from suggesting that these anomalies could be due to a hitherto unknown structural phase transition in coronene, the reasons for these deviations remained unknown. Over the years, similar anomalies were found when measuring the physical properties of coronene at low temperature^19^,^20^,^21^,^22^. Without rigorous explanation of this anomalous behaviour, the use of coronene as a valid model for the physical properties of graphene and for all of the predicted properties gleaned from computational modelling cannot be justified. Our recent work^23^ has revealed a previously unknown enantiotropic polymorph of coronene (β-coronene) Fig. 1(b). The structure of this new polymorph manifests itself with a significant shift to the π-stacking offset (*d*_*off*_ γ = 3.146 Å and *d*_*off*_ β = 1.606 Å) of molecules within the crystal [Fig. 1(c,d)] with negligible change to the stacking distance (γ = 3.484 Å, β = 3.467 Å). Here we show, through magnetic and structural characterisations, that the physical properties of the β-coronene polymorph and its spontaneous formation below 150 K, explain all of the previously observed anomalous behaviours of coronene at low temperature.

## Results

**Figure 1:**
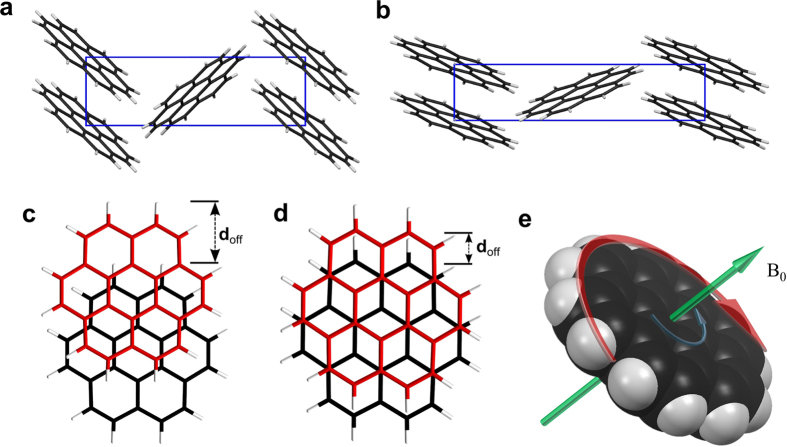
Representation of the (**a**) γ- and (**b**) β- forms of crystalline coronene as viewed along the a-axis. The difference in molecular overlap down the stack for γ- and β- are shown in (**c**,**d**) respectively and the calculated ring currents induced by a magnetic field are shown in (**e**). The larger diatropic current is shown as a red arrow and a smaller paratropic current in blue. Adapted from ref. 23.

**Figure 2:**
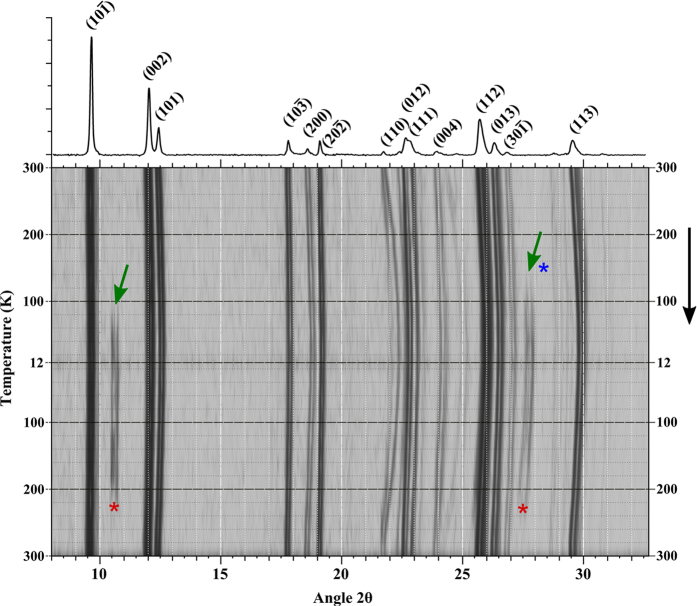
A 2D representation of powder diffraction data collected as a function of temperature in coronene. The top is an indexed, γ-coronene powder pattern to indicate peak positions. Temperature is decreased from 300 K to 12 K and then warmed back to 300 K (top to bottom). Green arrows indicate the emergence of the additional reflections due to β-coronene. Asterisks indicate the temperature at which the β-phase becomes detectable (blue) and drops back into the noise (red). Data in the top half of the figure is reproduced from ref. 23 with permission.

The discoidal nature of coronene coupled with a large π-system, make the molecule exquisitely susceptible to counter-rotating ring currents under an applied magnetic field [Fig. 1(e)] 24. Evidence of this phenomenon can be seen with an abnormally large chemical shift of the deshielded peripheral hydrogens, during ^1^H-NMR^25^. This gives the molecule a high magnetic anisotropy under an applied field which allows SQUID magnetometry to elucidate the difference in magnetic susceptibility of the two polymorphs as a function of the molecular rearrangement. Figure 2 shows the change in crystal structure as a function of temperature in γ-coronene. Temperature is shown decreasing from 300 K to 12 K and then warming back to 300 K (vertical axis). The top of the figure shows a single powder pattern from γ-coronene for clarity. In the box below, the peak height in counts is displayed in grey-scale (darker and lighter indicating a higher and lower number of counts respectively), and degrees 2θ from 8° to 32° is displayed from left to right. The β-coronene polymorph (approx. 3.6% of total crystal volume at 12 K, calculated via multi-phase Rietveld analysis) can be seen forming as a result of cooling, with the emergence of three peaks at 2θ = 10.46°, 10.69° and 27.78° at low temperature, which represent the (002), (101) and (112) reflections of β-coronene respectively. These peaks disappear and re-appear consistently with repeated heating and cooling cycles. The first appearance, during cooling, of reflections due to β-coronene; (002) at 100 K, (101) at 100 K and (112) 143 K, are marked on Fig. 2 with green arrows and the point at which they are no longer detectable, during warming, of (002) at 234 K, (101) at 234 K and (112) at 254 K marked with red asterisks. The blue asterisk denotes the highest temperature at which the β-coronene phase is detectable. At the lowest temperature (12.4 K) they have the relative intensity values (002) = 2.80%, (101) = 2.39% and (112) = 6.89%. As temperature decreases, the γ- unit cell contracts in a fairly linear fashion with the *c*-axis reaching a minimum at 74 K followed by the *a*- and *b*-axes reaching a minimum at 51 K. Upon warming, expansion also appears fairly linear, starting almost immediately on heating with no detectable thermal lag. Analysis of the unit cell over this temperature range shows an obvious hysteresis of the *a, b, c* and β parameters (Supplementary Information Figs 1, 2 and 3). Interestingly, the resulting unit cell volume, on warming, is observed to actually exceed that of the initial room temperature value above 250 K before contracting back to the original value at 300 K. This behaviour is likely to be a direct result of the crystal structure accommodating the interchange from γ to β but will require further investigation.

**Figure 3:**
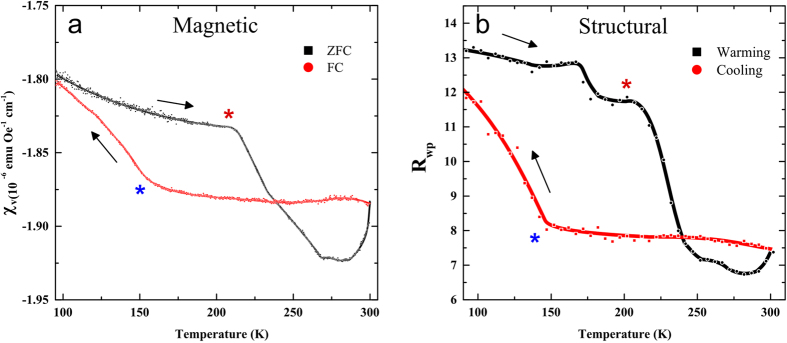
SQUID magnetometry and powder x-ray data for polycrystalline coronene. (**a**) Shows χ_v_ at 10 kOe and (**b**) shows the weighted R value (R_wp_) which is a measure of the goodness of fit of powder diffraction patterns, both as a function of temperature. On both graphs, the red and black markers represent data collected on cooling and warming respectively. The black arrows indicate the direction of the temperature change. The red and blue asterisks indicate the approximate positions of the emergence and disappearance, respectively, of the visible β-coronene peaks from the x-ray data in Fig. 2 and are at the same temperatures on both graphs. The added lines are a guide for the eye. The magnetic contributions of gel capsules and straws were subtracted from the signals.

Recognising the onset of the β-phase at reduced temperatures allows for a more precise interpretation of other data collected in a cryogenic environment. Using a SQUID magnetometer, the bulk magnetic susceptibility of the sample can be measured as a function of temperature and therefore of crystal structure. Magnetometry data for coronene show an apparent thermal hysteresis in magnetic susceptibility between 300 K and 100 K (Fig. 3). Upon increasing the temperature after applying a field, the sample becomes more diamagnetic and follows a curve that begins to stabilise at *χ*_*v*_ = −1.82 × 10^−6^ emu Oe^−1^ cm^−3^. A sharp increase in diamagnetism is observed at 212 K until 267 K where it remains mostly constant until 286 K, before reducing to *χ*_*v*_ = −1.88 × 10^−6^ emu Oe^−1^ cm^−3^ at 300 K. With the field still applied and the temperature decreasing, there is no change in susceptibility until 164 K where the signal decreases in diamagnetism until it intercepts the zero field cooled (ZFC) line at *χ*_*v*_ = −1.80 × 10^−6^ emu Oe^−1^ cm^−3^ at 98 K. The magnetic response of coronene at applied fields of *H*_0_ = 100 Oe, 10 kOe, 20 kOe and 50 kOe, shows that the magnetisation of coronene scales linearly with the applied field (Table 1). When comparing these results, it is clear that the crystallographic structural hysteresis has strikingly similar features to the thermal hysteresis observed in the SQUID measurements, and at similar temperatures (Fig. 3). The peaks appearing in the x-ray pattern at 150 K upon cooling (marked with a blue asterisk), clearly coincide with a marked decrease in diamagnetic susceptibility as seen in the magnetometry results. Upon warming, there are three points of note, 212 K, 267 K and 286 K. As the x-ray peaks begin to vanish at 212 K, SQUID magnetometry shows an increase in diamagnetic susceptibility until 267 K where susceptibility stabilises as the x-ray peaks diminish to below the level of noise in the pattern. Interestingly, the diamagnetic susceptibility is observed to surpass that of the initial cooling value significantly. When compared to the structural data, particularly the *b*-axis, this larger susceptibility coincides with the unexpected increase in the unit cell parameters upon warming, suggesting that a larger unit cell results in a larger diamagnetic susceptibility. The third point at ~286 K shows a sharp decrease in diamagnetic susceptibility before reaching the maximum temperature of 300 K. Upon re-cooling, the susceptibility does not decrease but remains relatively constant before an upturn towards the paramagnetic region at approximately 150 K. Figure 3 shows the magnetometry data with the positions of relevant peak activity marked with asterisks and the direction of thermal cycling marked with arrows. It would appear that the emergence and waning of the β-phase, sign-posted by the emerging reflections in the x-ray experiment, is having a direct effect on the magnetic behaviour of coronene. As there is no applied magnetic field present for the x-ray diffraction measurements, this shows unambiguously that at higher temperatures, the magnetic susceptibility thermal hysteresis is due to the structural transition from the γ- to the β-polymorph. As the hystereses in both the SQUID data and the powder diffraction correlate, it is reasonable to postulate that the new polymorph is having a discernible effect on how the coronene molecules respond to an applied magnetic field. When crystallographic reflections due to β-coronene are present, the overall bulk diamagnetic susceptibility is diminished. As this magnetic response scales with applied field to at least 20 kOe, this implies that the new phase is enhancing the paratropic ring current. In PAHs, a deviation from planarity in individual molecules reduces aromaticity^26^, so the reduction in thermal undulation at low temperature would cause an increase in ring currents under a constant field. It is therefore reasonable to assume that the minor reorganisation in crystal structure involving a unit cell contraction and deviation from planarity in the formation of β-coronene is the cause of this magnetic behaviour.

The discovery of the β-polymorph at temperatures below 100 K and the work presented here, allows us to resolve the previously reported anomalous data for low temperature coronene. Firstly, solid-state NMR on crystalline coronene at temperatures as low as 5 K observed an unexpected deviation of the δ_33_ tensor component of the ^13^C signal, from the axis perpendicular to the plane of the molecule^15^. This 13.4° offset at 5 K, not observed at room temperature, was suggested by the authors to be due to an unexpected axis of freedom for the molecules in the solid state at low temperatures. However, reassessment of this data with knowledge of the β-polymorph of coronene reveals that a partial transformation of the powder into β-coronene at low temperatures would evidently mean that two phases would be detected via solid-state NMR^27^. It is therefore clear that as γ-coronene and β-coronene differ only in molecular overlap and nearest neighbour angle, a mixture of the two polymorphs in the solid state could indeed be calculated as an average 13.4° shift in δ_33_ tensor. Quantitatively, based on a 35° difference in the nearest neighbour angle in the new polymorph we calculated that of the total mass of coronene tested in this previous study, approximately 38% assumed the β-form. We have observed higher percentages such as this in fast-cooled (shattered) or milled mixtures of γ-coronene and β-coronene, implying crystallite size is also a factor governing the proportion of crystals undergoing the transition^28^.

Secondly, regarding the optical response of coronene, our previous work has already established that the way in which each polymorph interacts with light differs significantly^23^. It would therefore be likely that this difference would have been observed in previous spectroscopic studies of coronene at low temperatures. Indeed the luminescence of coronene has been reported previously and in all studies, anomalous behaviour was reported to be observed below 90 K. Two papers reported on this transition in 1994 (refs 18 and 19) and made reference to a possible electronic or structural phase transition. These studies showed that changes in spectra from 298 to 2 K indicated the emergence of an absorbance band (band C, Fig. 2 from ref. 14) at 2 K that is a third the intensity of the expected 0-phonon band for the γ-polymorph. This band sits to the lower energy side, which would be expected from a greater overlap of the π-orbitals; precisely what is seen in the crystal structure of β-coronene. In addition to this, the increase in charge transfer excitons would see a drop in self-trapped excitons, especially within the ^1^L_b_ (separating from ^1^L_a_) band which is parallel to the *b*-axis, exhibiting the greatest overlap. Band C’s intensity at 2 K would again indicate >30% conversion into the β-polymorph.

Lastly, perhaps the most important sphere in which knowledge of the precise physical behaviour of coronene is essential, is in the field of computational chemistry. As stated earlier, coronene is a key molecule used for understanding behaviour and prediction of extended sp^2^ carbon systems including molecular crystals and graphene. In preparation of an analytical or predictive simulation using quantum chemical calculations, it is necessary to bench-mark all input parameters against experimental data. This bench-marking optimises the balance between quantum chemical calculations and the use of approximations, especially necessary when examining larger systems of periodic boundary conditions. The reason that this is critical is that the correct correlation of input to experimental data minimises computational load whilst validating further calculation within the same system. As we have shown in this work however, all semi-empirical calculations (and all subsequent research conducted using these methods), parameterised to adopt the assumed γ-coronene structure at cryogenic temperatures will be erroneous, due to the appearance of the β-coronene polymorph. More specifically, as we have shown that crystallographically the stacked molecular overlap in β-coronene (a geometry which most dispersion-corrected methods agree to be the most thermodynamically favourable) leads to a weakening of the C-H-π interaction, it follows that calculations on orbital interactions within crystalline coronene must take into account the appearance of the new polymorph in order to be considered to be accurate.

## Conclusions

In this work we demonstrate for the first time, the magnetocrystalline behaviour of the newly-discovered polymorph of coronene and show that the electronic behaviour of the material is intimately linked to the crystal structure as it changes from one polymorph to another under thermal cycling. In addition, we have shown that the previously unaccountable behaviour of coronene in spectroscopic studies (absorption, fluorescence and luminescence), along with ^13^C solid-state NMR and computational modelling is due to the appearance of the β-coronene polymorph at temperatures below 110 K and the concomitant change in physical properties below that temperature. From our work presented here, it is clear that in the future, the physical properties of the new β-coronene polymorph are taken into account in order to have confidence that low-temperature physical characterisations and optimised geometry calculations are correct.

## Methods

Coronene (97%) was purchased from Sigma-Aldrich UK and purified by sublimation under vacuum after recrystallization from toluene. All Rietveld analyses were carried out using Profex 3.9.2 software utilising the BGMN engine^29^. Both phases were refined as textured, due to a preference for growth along the *b*-axis intrinsic with the needle-like morphology of the crystals. A summary of results and refined cell parameters can be found in Supplementary Information Table 1.

### Crystallography

Variable temperature powder X-ray diffraction data were acquired using a Bruker D8 Advance diffractometer with a PSD LynxEye Detector and Oxford Cryosystems pHeniX cryostat (Cu-Kα radiation wavelength of 1.5418 Å). Step size was 0.0411°/2θ and step hold time was 1.5 s. Patterns were constantly collected as the temperature was repeatedly cycled from 300 K to 12 K.

### Magnetometry

Magnetic susceptibility measurements were taken with a Quantum Design, 7 T SQUID magnetometer. The magnetic susceptibility (20 mg of sample in gel capsules within plastic straws) was measured as a function of temperature using a ramp of 2 K min^−1^. Zero Field-Cooled (ZFC) measurements involved initially cooling the sample to 4.2 K before applying a magnetic field and measuring the magnetisation during warming at a constant rate. Field-Cooled (FC) measurements were taken by applying a magnetic field at the highest measured temperature (300 K) and recording the magnetisation whilst cooling at a constant rate.

### Data Availability

Raw data from X-Ray diffraction and SQUID magnetometry, pertaining to all materials in this manuscript have been placed in the University of Bristol Research Data Repository (https://data.bris.ac.uk/data/dataset/b5jhzznyczhm13lh9dhv8zy6w).

## Additional Information

**How to cite this article**: Potticary, J. *et al*. Low temperature magneto-morphological characterisation of coronene and the resolution of previously observed unexplained phenomena. *Sci. Rep.* **6**, 38696; doi: 10.1038/srep38696 (2016).

**Publisher's note:** Springer Nature remains neutral with regard to jurisdictional claims in published maps and institutional affiliations.

## Supplementary Material

Supplementary Information

## Figures and Tables

**Table 1:** Magnetic susceptibility values for coronene at 50 K, 150 K and 300 K under applied fields of 100 Oe, 10 kOe, 20 kOe and 50 kOe.

**H_0_ (Oe)	χ_v_ (10^−6^ emu Oe^−1^ cm^−3^)		
100	−0.02 ± 0.001	−0.02 ± 0.001	−0.02 ± 0.001
10000	−1.6 ± 0.05	−1.9 ± 0.05	−1.9 ± 0.05
20000	−3.2 ± 0.09	−3.6 ± 0.1	−3.6 ± 0.1
50000	−9.7 ± 0.3	−9.9 ± 0.3	−10.0 ± 0.3

## References

[b1] Moffitt W. E. & Coulson C. A. The Electronic Structure and Bond Lengths of Coronene and Pyrene. Proc. Phys. Soc. 60, 309–315 (1948).

[b2] Darvish G. M., Hosseini-khah S. M. & Amini-tabar Z. Theoretical insight into hydrogen adsorption onto graphene: a first-principles B3LYP-D3 study. Phys. Chem. Chem. Phys. 17, 2504–2511 (2015). 25490973 10.1039/c4cp04399e

[b3] Zhao Y. & Truhlar D. A Prototype for Graphene Material Simulation: Structures and Interaction Potentials of Coronene Dimers. J. Phys. Chem. C. 112, 4061–4067 (2008).

[b4] Rubio M., Enrique O. & Sánchez-Marín J. A study of coronene—coronene association using atom—atom pair potentials. Int. J. Quantum Chem. 57, 567–573 (1996).

[b5] Yoshida Y. . Conducting π Columns of Highly Symmetric Coronene, The Smallest Fragment of Graphene. Chem. Eur. J. 22, 6023–6030 (2016). 26989854 10.1002/chem.201505023

[b6] Yuan Y. L., Chen P. Y., Yang L. H., Ju Y. & Wang H. M. Quantum chemical insight into the reactivity of 1,3-dipoles on coronene as model for nanographenes. Russ. J. Phys. Chem. A. 90, 173–182 (2016).

[b7] Li J. W. . Describing curved-planar π-π interactions: modeled by corannulene, pyrene and coronene. Phys. Chem. Chem. Phys. 15, 12694–12701 (2013). 23793112 10.1039/c3cp51095f

[b8] Ma J., Michaelides A. & Alfe D. Binding of hydrogen on benzene, coronene, and graphene from quantum Monte Carlo calculations. J. Chem. Phys. 134, 134701 (2011). 21476763 10.1063/1.3569134

[b9] Zhechkov L., Heine T. & Seifert G. Physisorption of N_2_ on graphene platelets: An ab initio study. Int. J. Quantum Chem. 106, 1375–1382 (2006).

[b10] Federov I., Zhuravlev Y. & Berveno V. Properties of crystalline coronene: Dispersion forces leading to a larger van der Waals radius for carbon. Phys. Status Solidi B. 249, 1438–1444 (2012).

[b11] Diez-Perez I. . Gate-controlled electron transport in coronene as a bottom-up approach towards graphene transistors. Nature Commun. 1, 31 (2010). 20975686 10.1038/ncomms1029

[b12] Kubozono Y. . Metal-intercalated aromatic hydrocarbons: a new class of carbon-based superconductors. Phys. Chem. Chem. Phys. 13, 16476–16493 (2011). 21850291 10.1039/c1cp20961b

[b13] Kato T., Yoshizawa K. & Yamabe T. Jahn–Teller effects in the coronene anions and cations. J. Chem. Phys. 110, 249–255 (1999).

[b14] Todorov P. D., Jenneskens L. W. & van Lenthe J. H. Assignment of phantom bands in the solid-state infrared and Raman spectra of coronene: The importance of a minute out-of-plane distortion. J. Chem. Phys. 132, 034504 (2010). 20095745 10.1063/1.3282331

[b15] Fawcett J. K. & Trotter J. The Crystal and Molecular Structure of Coronene. Proc. R. Soc. A. 289, 366–376 (1966).

[b16] Zhao X. M. . Phase transformations and vibrational properties of coronene under pressure. J. Chem. Phys. 139, 144308 (2013). 24116620 10.1063/1.4824384

[b17] Ohno K., Inokuchi H. & Kajiwara T. Vibrational analysis of electronic transition bands of coronene. Bull. Chem. Soc. Jpn. 45, 996–1004 (1972).

[b18] Yamamoto T. . Exciton—phonon coupling and pressure-induced structural phase changes in coronene crystals. Chem. Phys. 184, 247–254 (1994).

[b19] Nakatani S., Nakamura T., Mizuno K. & Matsui A. H. Interband and intraband exciton scattering in corenene crystal. Journal of Luminescence. 58, 343–346 (1994).

[b20] Orendt A. M. . Carbon-13 Shift Tensors in Polycyclic Aromatic Compounds. J. Phys. Chem. A. 104, 149–155 (2000).

[b21] Totoki R., Aoki-Matsumoto T. & Mizuno K. Density of states of the lowest exciton band and the exciton bandwidth in coronene single crystals. J. Lumin. 112, 308–311 (2005).

[b22] Wong W. K. & Westrum E. F. Thermodynamics of Polynuclear Aromatic Molecules: II. Low-Temperature Thermal Properties of Perylene, Coronene, and Naphthacene. Mol. Cryst. Liq. Cryst. 61, 207–228 (1980).

[b23] Potticary J. . An unforeseen polymorph of coronene by the application of magnetic fields during crystal growth. Nature Commun. 7, 11555 (2016). 27161600 10.1038/ncomms11555 PMC4866376

[b24] Steiner E., Fowler P. W. & Jenneskens L. W. Counter-Rotating Ring Currents in Coronene and Corannulene. Angew. Chemie Int. *Ed.* 40, 362–366 (2001). 10.1002/1521-3773(20010119)40:2<362::AID-ANIE362>3.0.CO;2-Z 29712401

[b25] Jonathan N., Gordon S. & Dailey B. P. Chemical Shifts and Ring Currents in Condensed Ring Hydrocarbons. J. Chem. Phys. 36, 2443–2448 (1962).

[b26] Matito E., Poater J., Duran M. & Solà M. An analysis of the changes in aromaticity and planarity along the reaction path of the simplest Diels–Alder reaction. Exploring the validity of different indicators of aromaticity. J. Mol. Struct. Theochem 727, 165–171 (2005).

[b27] Byrn S. R., Gray G., Pfeiffer R. R. & Frye J. Analysis of solid-state Carbon-13 NMR spectra of polymorphs (benoxaprofen and nabilone) and pseudopolymorphs (cefazolin). J. Pharm. Sci. 74, 565–568 (1985). 2991492 10.1002/jps.2600740516

[b28] Li D. . Size-dependant phase transition in methylammonium lead iodide perovskite microplate crystals. Nature Comm. 7, 11330 (2016). 10.1038/ncomms11330 PMC4844678 27098114

[b29] Döbelin N. & Kleeberg R. Profex: a graphical user interface for the Rietveld refinement program BGMN. J. Appl. Crystallog. 48, 1573–1580 (2015). 10.1107/S1600576715014685 PMC4603273 26500466

