# Supplementary material for: Low temperature magneto-morphological characterisation of coronene and the resolution of previously observed unexplained phenomena
Source: Sci Rep. 2016 Dec 7;6:38696. doi: 10.1038/srep38696 (PMC5141578; doi:10.1038/srep38696)
Supplement: Supplementary Information [file srep38696-s1.doc]

**Low temperature magneto-morphological characterisation of coronene and the resolution of previously observed unexplained phenomena**

**Supplementary Information**

**Authors:** Jason Potticary, Rebecca Boston, Liana Vella-Zarb, Alex Few, Christopher Bell,

Simon R. Hall

**Supplementary Information Figure 1. Change in unit cell volume as a function of temperature.** Red and black markers represent data collected on cooling and warming respectively. The blue box highlights where the volume exceeds the initial size and the green arrow indicates the temperature of the ‘cross-over’ point in the magnetometry data.

**Supplementary Information Figure 2. Change in unit cell parameters as a function of temperature.** Red and black stars (*a*-axis), circles (*b*-axis) and triangles (*c*-axis) represent data collected on cooling and warming respectively. The blue box highlights where the parameters exceed the initial sizes and the green arrow indicates the temperature of the ‘cross-over’ point in the magnetometry data.

**Supplementary Information Figure 3. Change in unique monoclinic angle, β, as a function of temperature.** Red and markers represent data collected on cooling and warming respectively. This data clearly shows the structural hysteresis involved in the thermal cycling.

|  | *a* (Å) | *b* (Å) | *c* (Å) | β (°) | Fraction (%) |
| --- | --- | --- | --- | --- | --- |
| Gamma | 9.971 | 4.628 | 15.503 | 106.661 | 96.4 |
| Beta | 10.386 | 3.789 | 17.214 | 96.800 | 3.6 |

R_exp_ = 4.358

R_wp_ = 6.780

**Supplementary Information Table 1. Results of multiphase Rietveld analysis at 12 K.** Unit cell parameters and volume fraction of the γ- and β-phases in the powder. R_exp_ and R_wp_ represent the theoretical best fit and the actual fitting values respectively.
